# P146: Hands up. Improving hospital hand hygiene compliance rates as a key patient safety and quality initiative

**DOI:** 10.1186/2047-2994-2-S1-P146

**Published:** 2013-06-20

**Authors:** P Raggiunti, J Somani, S Peczeniuk, N Smith, S Fotheringham

**Affiliations:** 1 Infection Control, Rouge Valley Health System, Toronto, Canada

## Introduction

In 2009, the Ministry of Health and Long-Term Care mandated Ontario hospitals to begin reporting compliance for the four moments of hand hygiene. Rouge Valley Health System (RVHS) discovered it had very poor baseline results (2008–09): 40% for the first moment and 51% for the fourth moment. RVHS responded by making hand hygiene compliance a corporate priority, with the goal of attaining sustained hospital compliance rates of >90% for the first and fourth moments. To achieve this, RVHS put into action its Hands Up strategy.

## Objectives

Education: Deliver hand hygiene education to staff working in all inpatient care areas; Align education with the Ministry’s Just Clean Your Hands program, and focus training on proper hand hygiene technique.

Accountability: Establish a team for each inpatient unit responsible for conducting compliance audits and providing support; Report results monthly at all levels of the organization.

Culture: Establish staff, physicians and volunteers as the “face” of a promotional campaign, and disseminate campaign widely across many channels; Build staff buy-in through the use of innovative vehicles and events.

## Methods

Strengthening education: The foundation for hand hygiene compliance is education. Staff need to be aware of the required practice and how it should be performed. At Rouge Valley, multiple channels were offered for staff to receive information and training:

Establishing accountability: Putting education into action on a sustained basis requires a defined level of accountability. At Rouge Valley, this was achieved by putting in place a system that ensured accountability for each inpatient unit.

Creating a culture shift: Culture plays a significant part in hand hygiene compliance. It establishes what the expected practice is, and reinforces this behaviour over time. Rouge Valley has been able to develop a rich hand hygiene culture by fostering it at the grassroots level.

## Results

RVHS hand hygiene rates have vastly improved since the launch of the Hands Up strategy. For fiscal 2011-12 and 2012-13, RVHS has surpassed the provincial average achieving target compliance with rates at or above 90% for both the first and fourth moments.

## Conclusion

The Hands Up Strategy has helped to achieve and sustain breakthrough hand hygiene performance at RVHS.

## Disclosure of interest

None declared

